# Pandemic-Related Changes in Technology Use Among a Sample of Previously Hospitalized Older Adult New Yorkers: Observational Study

**DOI:** 10.2196/41692

**Published:** 2023-03-29

**Authors:** Brittany F Drazich, Ji Won Lee, Kathryn H Bowles, Janiece L Taylor, Shivani Shah, Barbara Resnick, Nayeon Kim, Sarah L Szanton

**Affiliations:** 1 School of Nursing University of Maryland Baltimore, MD United States; 2 School of Nursing Johns Hopkins University Baltimore, MD United States; 3 School of Nursing University of Pennsylvania Philadelphia, PA United States; 4 Center for Home Care Policy & Research VNS Heath New York City, NY United States; 5 School of Public Health Johns Hopkins University Baltimore, MD United States

**Keywords:** older adults, technology, COVID-19, well-being, elderly population, technology use, physical disability, virtual health, social interaction, digital gaming, digital learning

## Abstract

**Background:**

The COVID-19 pandemic increased the importance of technology for all Americans, including older adults. Although a few studies have indicated that older adults might have increased their technology use during the COVID-19 pandemic, further research is needed to confirm these findings, especially among different populations, and using validated surveys. In particular, research on changes in technology use among previously hospitalized community-dwelling older adults, especially those with physical disability, is needed because older adults with multimorbidity and hospital associated deconditioning were a population greatly impacted by COVID-19 and related distancing measures. Obtaining knowledge regarding previously hospitalized older adults’ technology use, before and during the pandemic, could inform the appropriateness of technology-based interventions for vulnerable older adults.

**Objective:**

In this paper, we 1) described changes in older adult technology-based communication, technology-based phone use, and technology-based gaming during the COVID-19 pandemic, compared to before the COVID-19 pandemic and 2) tested whether technology use moderated the association between changes in in-person visits and well-being, controlling for covariates.

**Methods:**

Between December 2020 and January 2021 we conducted a telephone-based objective survey with 60 previously hospitalized older New Yorkers with physical disability. We measured technology-based communication through three questions pulled from the National Health and Aging Trends Study COVID-19 Questionnaire. We measured technology-based smart phone use and technology-based video gaming through the Media Technology Usage and Attitudes Scale. We used paired *t* tests and interaction models to analyze survey data.

**Results:**

This sample of previously hospitalized older adults with physical disability consisted of 60 participants, 63.3% of whom identified as female, 50.0% of whom identified as White, and 63.8% of whom reported an annual income of $25,000 or less. This sample had not had physical contact (such as friendly hug or kiss) for a median of 60 days and had not left their home for a median of 2 days. The majority of older adults from this study reported using the internet, owning smart phones, and nearly half learned a new technology during the pandemic. During the pandemic, this sample of older adults significantly increased their technology-based communication (mean difference=.74, *P*=.003), smart phone use (mean difference=2.9, *P*=.016), and technology-based gaming (mean difference=.52, *P*=.030). However, this technology use during the pandemic did not moderate the association between changes in in-person visits and well-being, controlling for covariates.

**Conclusions:**

These study findings suggest that previously hospitalized older adults with physical disability are open to using or learning technology, but that technology use might not be able to replace in-person social interactions. Future research might explore the specific components of in-person visits that are missing in virtual interactions, and if they could be replicated in the virtual environment, or through other means.

## Introduction

Beginning with New York City as the COVID-19 epicenter in the United States (March-May 2020) [1,2], the pandemic generated morbidity and mortality numbers that had been unseen for a century. By the end of 2020, the Centers for Disease Control and Statistics ranked COVID-19 as the third leading cause of death, second only to heart disease and cancer [3]. Older adults, especially those with multimorbidity or existing deconditioning, have shouldered a disproportionate burden of the illness and death caused by COVID-19 [4-10].

In response to the COVID-19 pandemic, the first major public health strategy for disease containment was physical distancing, defined as maintaining space from others who are not within one’s household [8,11,12]. People who were aged 70 years and older or perceived themselves at an increased health risk reported greater adherence to physical distancing during the pandemic [13]. Numerous research studies have documented the psychosocial impact of such physical distancing measures for older adults such as increased depression, increased anxiety, increased loneliness, and decreased well-being [14-18].

In an effort to buffer the effects of COVID-19 distancing restrictions, many people maintained active lifestyles and social communication with others through technology-based platforms [19,20]. Examples of technology that were used to maintain activity engagement or social connection during the pandemic include web-based communication such as “video chat,” smartphone use such as reading the news, and web-based gaming. Historically, older adults have used novel technology less than younger populations [21-25]. However, the COVID-19 pandemic increased the importance of technology for all Americans, including older adults [26]. Although a few studies have suggested that older adults might have increased their technology use during the COVID-19 pandemic, further research is needed to confirm these findings, especially among different populations, and using validated surveys [26-31]. In particular, research on changes in technology use among previously hospitalized community-dwelling older adults, especially those with physical disability, is needed because older adults with multimorbidity and hospital-associated deconditioning were a population greatly impacted by COVID-19 and related distancing measures [4-10]. Obtaining knowledge regarding previously hospitalized older adults’ technology use, before and during the pandemic, could inform the appropriateness of technology-based interventions for vulnerable older adults.

For the purposes of this study, well-being was defined as a combination of emotional experiences (both positive and negative) of a person, as well as their life satisfaction [32]. Prior to the pandemic, research evidence on the association of technology use and the well-being of older adults had been inconsistent. Some studies indicated that novel technologies could help support the well-being of the aging population through facilitating social connection and optimizing their daily activities (eg, information gathering and health maintenance) [33-36]. Alternatively, other studies have suggested that such conclusions are overgeneralizations based on scarce evidence and poor study methodology [37,38]. Although further research is needed to examine this association, researchers must consider that the simple association of technology use and well-being could offer misleading findings when using data from the pandemic [28]. For example, older adults who had more stress and anxiety related to the pandemic were more likely to decrease their in-person visits with family and friends, and older adults who were more likely to decrease their in-person visits with family and friends were more likely to use technologies to maintain that connection [28]. Thus, examining a cross-sectional association between technology use and well-being, while not taking into account pandemic-related changes in in-person visits, might overstate the negative effects of technology use on older adults. Instead, research is needed to examine if older adults’ technology use changed the relationship between fewer in-person visits and well-being. Obtaining knowledge regarding the role of technology in buffering the emotional impact of distancing restrictions for previously hospitalized community-dwelling older adults is important because such research can inform future interventions that increase their technology use and access [39].

This study had two aims: (1) to describe changes in older adults’ technology-based communication, technology-based phone use, and technology-based gaming during the COVID-19 pandemic, compared to before the COVID-19 pandemic, and (2) to test whether technology use moderated the association between changes in in-person visits and well-being, controlling for demographics such as age, gender, income, and living alone status. For this study, we applied Galappatti and Richardson’s [40] Well-being Conceptual Framework, which describes the linkage between well-being and disaster risk reduction. According to this framework, disaster events can deplete the resources that help a person maintain their well-being. Elements that bolster well-being during a disaster are (1) social ecological factors, which include maintaining relationships and venues for engagement, (2) human capacity, which includes maintaining skills, knowledge, and a sense of identity, and (3) the material environment, which includes infrastructure and physical safety and comfort [40]. Consistent with this conceptual framework, older adults might have increased their technology use during the pandemic to maintain social ecological factors and human capacity. Additionally, those older adults who had high technology use during the COVID-19 pandemic, and thus bolstered their social ecological factors and human capacity, might have experienced higher well-being. Informed by Galappatti and Richardson’s [40] Well-being Conceptual Framework, we hypothesized that previously hospitalized older adults with physical disability increased their technology use during the pandemic, and that this technology use buffered the negative impact of decreased in-person visits on older adults’ well-being.

## Methods

### Sampling

CAPABLE (Community Aging in Place–Advancing Better Living for Elders) is a home-based intervention that addresses function through personalized goal-setting to improve the health and safety of older adults. We recruited participants from a research study based in New York City, conducted in collaboration with the Center for Home Care Policy & Research at VNS Health, testing whether CAPABLE decreases posthospitalization disability. Participants were eligible for the CAPABLE parent study if they were (1) aged 65 years or older, (2) within a 60-74–day period post hospital discharge, (3) discharged from postacute home health services, (4) able to stand up with or without assistance, (5) experiencing physical disability as determined by patient verbalization of difficulty with at least one activity of daily living (eg, difficulty walking or difficulty dressing), (6) not actively receiving radiation treatment or chemotherapy, (7) hospitalized 3 times or less in the last 12 months, (8) living in New York City for the next 5 months, and (9) cognitively intact, as determined by a score of ≥5 on the Callahan 6-item screener [41]. Participants were eligible for this substudy if they (1) were participants in the CAPABLE parent study and (2) received a score of ≥5 on the Callahan 6-item screener [41] at the time of the substudy’s interview survey. At the time of the substudy’s interview survey, participants were in a period between 13 and 28 months post hospitalization. In the order of participation in the CAPABLE parent study, we included the first 60 older adults who passed the cognitive screen and agreed to participate in this substudy.

### Data Collection

We collected data between December 2020 and January 2021, which was shortly after the peak of the COVID-19 pandemic in New York City. Considering the physical distancing measures instituted during this period, and some older adults’ reticence with computer use, we used a telephone-based objective survey. The researchers who surveyed participants (BFD and JWL) were trained in proper techniques for conversing with those with hearing impairment (eg, talking slower not louder and rewording). The survey contained 50 items, most of which were multiple-choice questions, and took approximately 40 minutes to complete. We audio-recorded all calls, which the first author (BFD) checked for accuracy.

### Ethical Considerations

The Johns Hopkins University’s institutional review board and VNS Health’s institutional review board approved this study (E17-002). Prior to participation, a research assistant (BFD or JWL) informed participants that the study participation was voluntary, and if they chose not to participate, their care at VNS Health would not be affected. All participants provided verbal informed consent and all participant data were deidentified. Participants were mailed a US $25 gift card for their study participation.

### Survey Measures

#### Technology Use

Although data for this study were collected at one time point, we asked participants about their technology use currently, as well as (retrospectively) prior to the pandemic, which allowed for the examination of changes in older adults’ technology use during the pandemic. Similar to the National Health and Aging Trends Study COVID-19 Questionnaire [28,42], we established March 2020 as the time point in which “the effects of the outbreak first began.” For the 2 time frames “before the pandemic” and “during the pandemic,” we measured 3 types of technology: technology-based communication, technology-based smartphone use, and technology-based video gaming. These 3 measures of technology use are described below.

We measured technology-based communication through 3 questions extracted from the National Health and Aging Trends Study COVID-19 Questionnaire and described by Drazich et al [28] and Freedman Vicki and Kasper [42]. These items assess the frequency of social communication with family and friends through various forms of technology: (1) telephone calls, (2) emails, texts, and social media, and (3) video calls. For each item, participants reported their weekly frequency using a 5-point scale from 0=“never” to 4=“at least daily.” Summed responses ranged from 0 to 12, with higher scores indicating the highest frequency of technology-based communication. These 3 technology-based communication questions were asked in relation to the period “during the COVID-19 pandemic” and (retrospectively) “before the COVID-19 pandemic.”

We measured technology-based smartphone use through the 9-item “Smartphone Usage Sub-Scale” within the Media Technology Usage and Attitudes Scale [43]. The Smartphone Usage Sub-Scale has a Cronbach α of .93 [43]. These items assess how often a participant uses his/her smartphone for 9 purposes: reading emails, getting directions, browsing the web, listening to music, taking pictures, checking the news, recording a video, using apps, or searching for information. For each item, participants respond using a 10-point frequency scale from 1=“never” to 10=“all the time.” Summed responses ranged from 9 to 90 with higher scores indicating the highest frequency of smartphone use. These 9 technology-based smartphone questions were asked in relation to the time period “during the COVID-19 pandemic” and (retrospectively) “before the COVID-19 pandemic.” Of note, the Smartphone Usage Sub-Scale was only administered to participants who responded “yes” to owning a smartphone.

We measured technology-based video gaming through the 3-item “Video Gaming Sub-Scale” within the Media Technology Usage and Attitudes Scale [43]. The Video Gaming Sub-Scale has a Cronbach α of .83 [43]. These items assess how often a participant plays games on his/her computer, video game console, or smartphone (1) by him-/herself, (2) with other people in the same room, and (3) with other people on the web. Again, for each item, participants respond using a 10-point frequency scale from 1 “never” to 10 “all the time.” For the Video Gaming Sub-Scale, summed responses ranged from 3 to 30, with higher scores indicating the highest frequency of video gaming. These 3 technology-based video gaming questions were asked in relation to the period “during the COVID-19 pandemic” and (retrospectively) “before the COVID-19 pandemic.”

For descriptive purposes, we asked whether participants had access to the internet (yes/no), owned a smartphone (yes/no), and whether they learned a new technology during the COVID-19 pandemic (yes/no) and why (open answer).

#### Change in in-Person Visits

We measured change in in-person visits through a question that asked, “In a typical week, how often have you been in contact through in-person visits with family and friends not living with you?” Participants reported 0=“never,” 1=“less than once a week,” 2=“about once a week,” 3=“a few times a week,” or 4=“at least daily.” We asked this question for 2 periods: “before the pandemic” (retrospective report) and “during the pandemic.” We then subtracted responses “before the pandemic” from “during the pandemic” to obtain the “change in in-person visits” score. This “change in in-person visits” score ranged from –4 to +4, with negative numbers indicating a decrease in in-person visits during the pandemic and positive numbers indicating an increase in in-person visits during the pandemic. For descriptive purposes, we also asked the participants to report the number of days since they last had physical contact with a person (friendly hug or kiss), and the number of days since they had left their house for any reason (walk, grocery store, and pharmacy).

#### Well-being

We measured well-being through the 4-item Personal Well-being Scale [44]. The Personal Well-being Scale has an interitem correlation coefficient of 0.77 and Cronbach α of .9 [44]. These items assess both the emotional components (“I was happy yesterday” and “I was not anxious yesterday”) and the life satisfaction components (“I am satisfied with my life” and “What I do in my life is worthwhile”) of well-being [32,44]. Participants responded using a 4-point Likert scale from “disagree” to “strongly agree.” “I was not anxious yesterday” was reverse coded and all items were summed for a range between 0 and 16, with higher scores indicating better well-being.

#### Covariates

We included 4 covariate variables that are associated with older adult technology use and were collected through self-report: age, gender, living alone status, and income. We measured age on a raw continuous scale (see Table 1). Gender and living alone status consisted of 2 nominal categories (male/female; lives alone/lives with others). We measured income through interval data with six categories ranging from US $5000-$9999 annually to US $100,000 or above annually, with higher scores indicating greater income.

### Statistical Analysis

We first conducted descriptive analyses for each variable, assessed measures of central tendency and outliers, as well as model assumptions. To fulfill the first study aim, which was to describe changes in technology use due to the COVID-19 pandemic, we performed paired *t* tests. To fulfill the second study aim, to test whether technology use significantly changed the relationship between changes in in-person visits and well-being, we tested an interaction model with technology use as the moderator. We considered a 1-sided *P* value of <.05 as statistical significance and performed statistical analyses using SPSS (version 26; IBM Corp).

## Results

### Sample

Our sample consisted of 60 participants, 63.3% of whom identified as female, and 50.0% of whom identified as White (see Table 1). The participants reported various chronic conditions, with a total of 14 (23.3%) reporting a history of heart attack, 24 (40%) reporting a history of asthma or wheezing, 16 (26.7%) reporting a history of colitis, and 13 (21.7%) reporting a history of cancer. The majority of participants (63.8%) had an annual income of US $25,000 or less and 46.7% of participants reported living alone. On a scale from 0-4, with 4 indicating the highest score for in-person visits, participants decreased their in-person visits from a score of 2.19 before the pandemic to a score of 1.49 during the pandemic (mean difference –70, *P*<.001). At the time of the survey between December 2020 and January 2021, participants reported that they had not had physical contact (such as friendly hug, kiss, or handshake) for a median of 60 days and had not left their home for a median of 2 days. A total of 10 (17%) participants in this sample reported that they had not left their home for more than 20 days. Approximately 54 (90%) participants reported having access to the internet and 40 (66.7%) participants reported owning a smartphone. A total of 28 (46.7%) participants reported “learning a new technology during the pandemic.” The most common type of technology learned was videoconferencing software for the purpose of socialization, health (eg, telehealth), or activity engagement (eg, religious service). The average well-being score, which ranges 0-16 with higher scores indicating higher well-being, was 11.03 (SD 3.4).

**Table 1:** Participant characteristics (N=60).

Characteristics		Value
Age (years), mean (SD)		75.9 (7.1)
**Age (years), n (%)**		
	65-69	12 (20)
	70-74	16 (27)
	75-79	12 (20)
	80-84	15 (25)
	85-89	4 (7)
	≥90	1 (2)
**Sex, n (%)**		
	Women	38 (63)
	Men	22 (37)
**Race or ethnicity, n (%)**		
	White	30 (50)
	African American	21 (35)
	Other	9 (15)
	Hispanic	8 (14)
**Income^a^ (US $), n (%)**		
	5000-9999	10 (17)
	10,000-14,999	11 (19)
	15,000-24,999	16 (28)
	25,000-34,999	7 (12)
	≥35,000	14 (24)
**Live alone status, n (%)**		
	Live alone	28 (47)
	Live with others	32 (53)
**Education^a^, n (%)**		
	More than high school	6 (10)
	High school	19 (33)
	Technical degree	5 (9)
	Associate’s or bachelor’s degree	16 (28)
	Graduate school	12 (21)

^a^A total of 2 participants refused to report their income and educational attainment.

### Aim 1: Change in Technology Use

Fulfilling aim 1, we first tested if older adults’ technology-based communication, technology-based smartphone use, and technology-based gaming increased during the COVID-19 pandemic, compared to before the COVID-19 pandemic. We found that our sample of 60 previously hospitalized older adults with physical disability significantly increased their technology-based communication (*P*=.003), smartphone use (*P*=.02), and technology-based gaming (*P*=.03) during the pandemic, compared to those before the pandemic (see Table 2). On a scale from 0 to 12, with higher scores indicating the highest frequency of technology-based communication, older adults increased their technology-based communication from 6.4 points to 7.1 points. In particular, older adults who responded “never” to frequency of video calls changed from 63.3% before the pandemic, to 44.1% during the pandemic. On a scale from 9 to 90, with higher scores indicating the highest frequency of smartphone use, older adults increased their smartphone use from 27.0 points to 30.0 points. On a scale from 3 to 30, with higher scores indicating the highest frequency of technology-based gaming, older adults increased their technology-based gaming from 5.2 points to 5.7 points.

**Table 2:** Change in technology use.

	Participants, n	Before the pandemic, mean (SD)	During the pandemic, mean (SD)	Change, mean (SD)	*P* value
Technology-based communication	57	6.4 (3.1)	7.1 (2.9)	.74 (2.0)	.003
Smartphone use	39	27.0 (11.3)	30.0 (12.4)	2.9 (8.1)	.02
Technology-based gaming	59	5.2 (3.5)	5.7 (3.6)	0.52 (2.1)	.03

### Aim 2: Moderation Effects of Technology Use

Fulfilling aim 2, we then tested whether technology use during the pandemic moderated the association between changes in in-person visits with family and friends and well-being, controlling for age, gender, income, and living alone status. We found that technology-based communication (b=–0.19, *P*=.17), technology-based smartphone use (b=–0.0002, *P*=.99), and technology-based video gaming (b=–0.03, *P*=.82) during the pandemic did not moderate the association between changes in in-person visits and well-being, controlling for covariates. Thus, the relationship between change in in-person visits and well-being is the same, regardless of level of technology use.

## Discussion

### Principal Findings

This sample of 60 previously hospitalized older adults with physical disability significantly increased their technology-based communication, smartphone use, and technology-based gaming during the pandemic, compared to before the pandemic. This finding complements previous research, which indicated that older adults increased their technology use during the pandemic [27,28,45]. This finding is distinct from previous findings in that it specifically examined smartphone use and web-based gaming using surveys with tested psychometric properties, and was in a sample of vulnerable older adults [43]. This study also found that technology use during the pandemic did not significantly moderate the association between changes in in-person visits and well-being.

The finding that previously hospitalized older adults with physical disability increased their technology use during the pandemic has research and clinical implications. First, although older adults use technology less than other age cohorts, this study suggests that older adults should not be thought of as non–technology users [21-25]. The majority of previously hospitalized older adults from this study reported using the internet and owning smartphones, and nearly half of them learned a new technology during the pandemic. Thus, researchers should consider that many older adults are open to using technology, and this should, therefore, be considered in the design and implementation of technology-based health interventions. Second, the finding that nearly half of this sample of previously hospitalized older adults with physical disability learned a new technology during the pandemic indicates that many vulnerable older adults might be open to learning new technology-based interventions.

This study was guided by Galappatti and Richardson’s [40] Well-being Conceptual Framework, which suggests that the following elements bolster well-being during a disaster: (1) social ecological factors, (2) human capacity, and (3) the material environment. Consistent with this framework, we hypothesized that older adults who had high technology use during the COVID-19 pandemic, and thus bolstered their social ecological factors and human capacity, would have higher associated well-being. The framework’s third element that contributes to well-being, the “material environment,” was not explored in this study, but might help explain our finding that technology use did not buffer the effects of changes in in-person visits on older adults’ well-being. The “material environment” includes infrastructure and the degree of physical safety and comfort. In this study, older adults who used technology could have had financial concerns related to data usage, internet security concerns, stress due to technology use confusion, or addictive technology tendencies. Greater research is needed to investigate the potential negative effects of technology on older adults such as problematic technology use and technostress [46,47].

The findings that technology use did not significantly buffer the effects of changes in in-person visits on older adults’ well-being was a surprising finding and can have implications far beyond the pandemic. Older adults are often unable to experience in-person interaction due to a variety of reasons, such as mobility limitations, transportation inaccessibility, or income restraints. This study indicates that technology might not provide or supplement the full benefits of in-person visits. Future research might explore the specific components of in-person visits that are missing in digital interactions, and if they could be replicated in the digital environment, or through other means. In particular, greater investment in social or “cuddly” robotics might be warranted in the field of geriatrics, especially robots with high usability and safety, and those that are designed to support the values of the individual users [48-51].

### Limitations

Given the small sample size of 60 participants, this study might not have been powered to find statistical differences. Additionally, this sample was drawn from a population of older adults who live in New York City and had already received the CAPABLE intervention, which addresses physical function through goal-setting. Thus, this sample might be different form the general population of recently hospitalized older adults with functional disability who have not received the CAPABLE intervention or who live in different geographic regions. Another study limitation was the data collection at one time point. Participants might have had difficulty retrospectively reporting on their technology use prior to the COVID-19 pandemic (recall bias). Conversely, a strength of this study was the inclusion of older adults who are underrepresented in geriatric research, such as adults older than 75 years, Black older adults, and low-income older adults.

### Conclusions

This study found that previously hospitalized older adults significantly increased their technology use during the pandemic, compared to before the pandemic, but this technology use did not significantly moderate the association between changes in in-person visits and well-being. These study findings suggest that older adults are open to using or learning technology, but that technology use might not be able to replace in-person social interactions. These findings can guide researchers and clinicians in the postpandemic environment for the planning of technology-based health interventions.

## Abbreviations

CAPABLE: Community Aging in Place–Advancing Better Living for Elders

## Data Availability

The data sets generated during and/or analyzed during the current study are available from the corresponding author on reasonable request.
